# Survivin Improves Reprogramming Efficiency of Human Neural Progenitors by Single Molecule OCT4

**DOI:** 10.1155/2016/4729535

**Published:** 2016-11-16

**Authors:** Shixin Zhou, Yinan Liu, Ruopeng Feng, Caiyun Wang, Sibo Jiang, Xiaoyan Zhang, Feng Lan, Yang Li

**Affiliations:** ^1^Department of Cell Biology and Stem Cell Research Center, School of Basic Medicine, Peking University, Beijing, China; ^2^Baotou Medical College, Baotou, Inner Mongolia, China; ^3^Beijing Cellapy Biotechnology Co., LTD, Beijing, China; ^4^Department of Pharmaceutics, University of Florida, 6550 Sanger Road, Orlando, FL 32827, USA; ^5^Beijing Anzhen Hospital, Beijing, China

## Abstract

Induced pluripotent stem (iPS) cells have been generated from human somatic cells by ectopic expression of four Yamanaka factors. Here, we report that Survivin, an apoptosis inhibitor, can enhance iPS cells generation from human neural progenitor cells (NPCs) together with one factor OCT4 (1F-OCT4-Survivin). Compared with 1F-OCT4, Survivin accelerates the process of reprogramming from human NPCs. The neurocyte-originated induced pluripotent stem (NiPS) cells generated from 1F-OCT4-Survivin resemble human embryonic stem (hES) cells in morphology, surface markers, global gene expression profiling, and epigenetic status. Survivin keeps high expression in both iPS and ES cells. During the process of NiPS cell to neural cell differentiation, the expression of Survivin is rapidly decreased in protein level. The mechanism of Survivin promotion of reprogramming efficiency from NPCs may be associated with stabilization of *β*-catenin in WNT signaling pathway. This hypothesis is supported by experiments of RT-PCR, chromatin immune-precipitation, and Western blot in human ES cells. Our results showed overexpression of Survivin could improve the efficiency of reprogramming from NPCs to iPS cells by one factor OCT4 through stabilization of the key molecule, *β*-catenin.

## 1. Introduction

Reprogramming of somatic cells into induced pluripotent stem (iPS) cells holds great promise for the personal regenerative medicines [1]. The injured human cells or tissues could be replaced by the similar differentiation cells from human iPS cells [2]. The method of genomic integration of transgenes increases the risk of tumor formation and other abnormal issues [3]. Nonintegration human iPS cells have been generated using adenoviral or plasmid transfection methods [4, 5]. Other DNA-free methods include Sendai virus [6], direct protein delivery, and modified mRNA or microRNA transformations [7]. In this study, considering the key transcription factor, SOX2, is endogenously highly expressed in human neural stem cells (NSCs) or progenitors (NPCs) in the process of reprogramming [8, 9], we applied an episomal vector [10], one of no genome integration method, to reprogram human NPCs to iPS cells with only one factor OCT4 (1F-OCT4).

The self-renewal maintenance of human pluripotent stem cells (PSCs)* in vitro* needs supports of growth factor (e.g., bFGF) and extracellular matrix (ECM). Better understanding of PSCs self-renewal makes it possible to develop the medium with defined chemicals. The chemical defined medium is a fully defined, feeder-free medium formulated for the growth and expansion of human PSCs [11]. For ECM element, the feeder-free matrigel, which is a gelatinous protein mixture secreted by mouse Engelbreth-Holm-Swarm (EHS) sarcoma cells, is widely used in cultivated human PSCs [12]. The disadvantage of this xeno-proteins originated from mouse EHS may cause antigen response when applying iPS cells in human regeneration medicine [13]. Here we use human originated vitronectin (Xeno-free) instead of matrigel as ECM for maintaining iPS pluripotency just for the safety concern.

There are some reports showing that iPS cells retain an epigenetic memory of their original tissues in mouse and human iPS cells [14]. Residual methylation signatures link iPS cells to their tissue of origin and even discriminate between the myeloid and lymphoid origins of blood-derived iPS cells [15, 16]. Incomplete DNA methylation indicates a transcriptional memory of somatic cells in human iPS cells, especially in the early passages [17]. All low-passage iPS cells analyzed retain a transcriptional memory of the original cells. Such a memory would be the fingerprint of the iPS cell's somatic origin [16]. iPS cells derived from human pancreatic islet beta cells demonstrated an increased ability to differentiate into insulin-producing cells, compared with ES cells and isogenic non-beta iPS cells [14]. All these evidences indicate that iPS cells originated from neural progenitors carved with epigenetic memory may benefit easier differentiating to neural cells.

Survivin is an important member of IAP (inhibitor of apoptosis) family; it functions as an apoptosis inhibitor in different types of cell especially in cancer cells. Survivin expression in normal tissue is developmentally regulated and has been reported to be low in most terminally differentiated tissues. But it has also been showed that Survivin also expressed in ES cell and NSCs (NPCs), OCT4, or SOX2 regulates its expression in those cells. Survivin expression is positively related to pluripotency maintenance of ES cells or iPS cells [18]. In our previous research, upregulation of Survivin could inhibit neural stem cells apoptosis mediated by SOX2 [19]. WNT signaling pathway reported plays an important role in promoting somatic cell reprogramming; the mechanism is that *β*-catenin interacts with reprogramming factors KLF4, OCT4, and SOX2 to enhance expression of pluripotency circuitry genes [20]. Here, we found overexpression of Survivin in NPCs could enhance the efficiency of 1F-OCT4 reprogramming to iPS cells. The mechanism of Survivin improving the efficiency of NPCs reprogramming may relate to WNT signaling pathway by stabilization of the key molecule, *β*-catenin. We found Survivin can directly interact with *β*-catenin in ES cells or iPS cells by immunoprecipitation (IP) analysis. Our results indicated that stabilization of *β*-catenin would benefit pluripotency maintenance and could enhance the efficiency of reprogramming.

## 2. Materials and Methods

### 2.1. Cell Isolation and Culture

Human embryonic cortical tissues were precisely dissected from naturally miscarried fetal brains at 66,74 and 90 days, respectively. The authorization for using human materials and the parent's informed consent was approved by the Ethics Committee of Peking University Health Science Center and carried out according to the guidelines of the World Medical Association Declaration of Helsinki (http://www.wma.net/en/30publications/10policies/b3/). Human fetal cortical tissue was treated with 0.25% trypsin for 15 minutes, washed with Dulbecco's minimal essential medium (DMEM; Hyclone Laboratories, Logan, UT, USA), and dissociated into a single cell suspension. The cells were seeded at a density of 100,000 cells/mL into T75 flasks containing 20 mL of serum free medium (DMEM : F12) supplemented with B27 (2%, Life Technologies, Carlsbad, CA, USA), EGF (20 ng/mL, Peprotech, Rocky Hill, NJ, USA), FGF2 (20 ng/mL, Peprotech, Rocky Hill, NJ, USA), and heparin (5 *μ*g/mL, Sigma, St. Louis, MO, USA). NPCs were passaged by a chopping method previously described [21]. One day before infection, NPCs were digested into single cells by Accutase (Sigma, St. Louis, MO, USA), and 100,000 cells were seeded to poly-ornithine-coated six-well plates to form a monolayer culture.

### 2.2. Episomal Vectors to Cells by Electroporation

The episomal vectors (EBNA-1) are a well-described system for producing transgene-free, virus-free iPS cells [10]. The episomal vector containing pCXLE-OCT4 (1F-OCT4) was introduced into human NPC by electroporation (Amaxa Nucleofector 2B, LabWrench, Canada) on day 0. The transfected cells were plated onto vitronectin-coated culture dishes. We used Reproeasy chemical defined medium (Cellapy, Beijing, China) to cultivate 1F-OCT transfected cells from day 1 to day 14. Mediums were changed to PSCeasy chemical defined medium (Cellapy, Beijing, China) from day 15 on vitronectin-coated culture vessels, using 0.5 mM EDTA as the passaging reagent, according to the manufacturer's instructions. Survivin knockdown shRNAs (CCAGTGTTTCTTCTGCTTCAA) were cloned into the adenovirus shuttle vector pDC316-EGFP with U6 promoter and full-length Survivin cDNA was cloned into the pDC316-EGFP vector. Recombinant adenovirus was generated using the AdMax system (MicrobixBiosystems). Adenovirus was purified using the ViraBindTM Adenovirus Purification Kit (Cell Biolabs). The NPCs were infected at a MOI (multiplicity of infection) of 60.

### 2.3. Immunofluorescence and Immunochemistry

Cells were grown on cover slides and fixed with 4% paraformaldehyde. The following antibodies to SOX2, NANOG, Nestin, SSEA-4, TRA-1-60, and TRA-1-81 were all purchased from Chemicon (Temecula, CA, USA) and anti-OCT4 was from Santa Cruz Biotechnology (Santa Cruz, CA, USA). Rabbit polyclonal antibody of Survivin was from Cell Signaling Technology, Inc. (China) and *β*-catenin antibody was from Sigma-Aldrich (St. Louis, MO, USA). The second antibodies (Alexa Fluor Series) were from Invitrogen (Grand Island, NY, USA). The cell AP activity was analyzed by an alkaline phosphatase blue membrane substrate solution kit (Sigma-Aldrich, St. Louis, MO, USA), according to the manufacturer's guidelines.

### 2.4. Gene Expression Profiling and Methylation Analysis

The global gene expression profiling was performed by Affymetrix Human U133 plus 2.0 microarrays (Affymetrix, Santa Clara, CA, USA). For transcriptome profiling, 5 *μ*g of total RNA was used as input for labeled cRNA synthesis following the manufacturer's instructions (IVT: 12 hours). Quality-checked cRNA samples were hybridized as biological for 16 hours onto Human U133 plus 2.0 3′ expression microarrays. All the steps including washing, staining, and scanning signals followed suggestions by the manufacturer. The microarray data were analyzed by Affymetrix Expression Console Software 1.3 (Affymetrix, Santa Clara, CA, USA). The analysis of MvA plot was used to compare two microarrays (cell types) identity. The method also used Pearson's correlation (*R* ^2^) value to indicate the similar. The hierarchical clustering analysis was carried out using Cluster 3.0 ([22], as updated by Michiel de Hoon) and Treeview software using mean-centered Pearson correlation and complete linkage. DNA methylation analysis on OCT4 and NANOG was described as Freberg et al. [23].

### 2.5. Luciferase Activity Assays

The Survivin promoters were amplified by PCR from human genome DNA, digested with XhoI and HindIII, and then ligated into the reporter vector pGL3-Basic (Promega, USA). The primers of Survivin promoter for PCR were forward, 5′AGCTCGGCGGGGTGGGAGGGGTGGGGAG3′, and reverse, 5′AGCTTAGTAGAGGCGGGGCGGCGCG3′. HEK 293 cells were seeded on a 24-well plate and transiently transfected with the Survivin promoter reporter vector and SOX2 or OCT4 expression plasmid using Lipofectamine 2000 (Invitrogen, USA). pRL-CMV (Promega, USA) expressing Renilla luciferase was transfected as an internal control. After 48 h, the transfected HEK293 cells were lysed in passive lysis buffer and luciferase activity was measured with the dual-luciferase reporter assay system (Promega, USA) using a Centro LB960 96-well luminometer (Berthold Technologies, Germany).

### 2.6. Chromatin Immunoprecipitation (ChIP) and Western Blot Analysis

To generate chromatin, about 1 × 10^7^ infected ES cells or iPS cells were collected and fixed with 1% polyformaldehyde. The formaldehyde was inactivated by the addition of 125 mm glycine. Cellular lysates were prepared by incubating the cells in lysis buffer (50 mM Tris-HCl, pH 8.0, 150 mM NaCl, 0.5% NP40) for 20 min at 4°C. The chromatin containing genomic DNA in lysis buffer was fragmented by sonication to the ranges of 150–500 bp. The fragmented chromatin is then precleared with protein A and G sepharose beads and immunoprecipitated with antibodies of interest and sepharose beads. The material is then washed, eluted, and purified with the Qiagen PCR purification kit. The samples are then analyzed by qPCR. ChIP assays were performed as described previously [8]. For PCR, 1 *μ*L of DNA extraction was used and amplified for 21–25 cycles. The primers of proximal promoter of Survivin were forward, 5′GACGCCCTGCTTTGCGAAGG3′, and reverse, 5′CTTCTGGAGTCAGGGGCCAGGG3′. Primers of NANOG promoter were forward, 5′GCTGGTTTCAAACTCCTGACTTC3′, and reverse, 5′CAACAGAACCTGAAGACAAAC3′. Primers of *β*-actin promoter were forward, 5′AGAAAATCTGGCACCACACC3′, and reverse, 5′CGAGCCATAAAAGGCAACTTTCGGA3′.

The RT-qPCR reactions were set up in triplicate with the SYBR Premix assay (Takara, Japan). Reactions were run on a Stratagene 3000P real-time PCR machine (Stratagene, USA) with 40 cycles of 30 s at 95°C, 30 s at 58°C, and 30 s at 72°C [24].

For Western blotting, cells were lysed with ice-cold lysis buffer and a protease inhibitor cocktail [19]. Equal amounts of extracted proteins (50–80 *μ*g) were resolved with SDS/PAGE (10% gels) electrophoresis; then the gel was transferred on to 0.2 *μ*m PVDF membranes. After blocking in 5% skimmed milk for 1 h, the membranes were incubated in the primary antibodies at 4°C overnight. The antibodies used were rabbit polyclonal anti-SOX2, anti-Survivin, and mouse monoclonal anti-*β*-actin. The membranes were washed with PBST buffer 3 times. After incubation in labeled fluorescent secondary antibodies (Rockland, USA) for 1 h at room temperature, images on membrane were developed with the Odyssey Western blotting system (LI-COR Bio, USA).

### 2.7. Reverse Transcription PCR (RT-PCR)

RNA was extracted using the RNeasy Plus Mini Kit (Qiagen, Germany) in accordance with the manufacturer's instructions. For reverse transcription-quantitative PCR (RT-qPCR), SYBR Premix assay (Takara, Japan) was used to perform real-time PCR with MX3000P. The following thermal profile was used for all PCR experiments: 95°C for 5 min; 40 cycles at 95°C for 30 s; annealing temperature 58–60°C for 30 s; and it is terminated by a final extension at 72°C for 10 min.

The primers used for RT-PCR were SOX2 forward, 5′GCCGAGTGGAAACTTTTGTCG3′, and reverse, 5′GGCAGCGTGTACTTATCCTTCT′3, and Survivin forward, 5′AGGACCACCGCATCTCTACAT3′, and reverse, 5′AAGTCTGGCTCGTTCTCAGTG3′.

### 2.8. ES (iPS) Cell Differentiation with Retinoic Acid (RA)

ES or iPS cells were detached and dissociated into single cells with 0.5 mM EDTA and then plated onto a bacteriological dish in 10 mL of a-MEM (Gibco) supplemented with 10% FBS, sodium bicarbonate (3 mM), and 0.1 mM 2-ME (EB medium) at a density of 5 × 10^4^ cells/mL. On day 2, Retinoic Acid (Sigma-Aldrich, St. Louis, USA) of 500 nM was added to the culture medium and maintained for 96 h [25]. The cells of each 24 h were collected for further analysis.

## 3. Results

### 3.1. The Reprogramming Process of Human NPCs with 1F-OCT4

**Figure 1:**
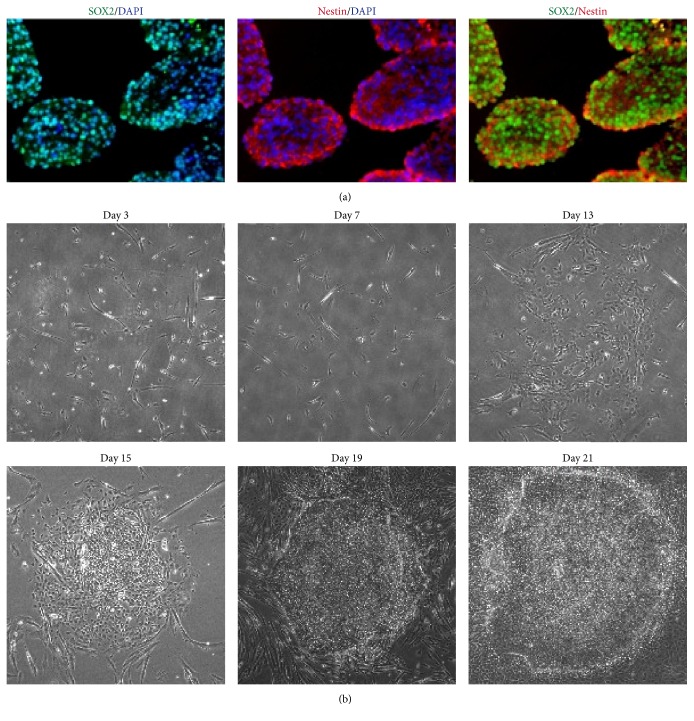
SOX2 and Nestin were endogenously expressed in human NSC or NPCs. (a) SOX2 (green) and Nestin (red) were endogenously expressed in human NSC or NPCs. Nuclei were stained with DAPI (blue). (b) Reprogramming process of NPCs under phase contrast microscopy. Scale bars, 50 *μ*m.

**Figure 2:**
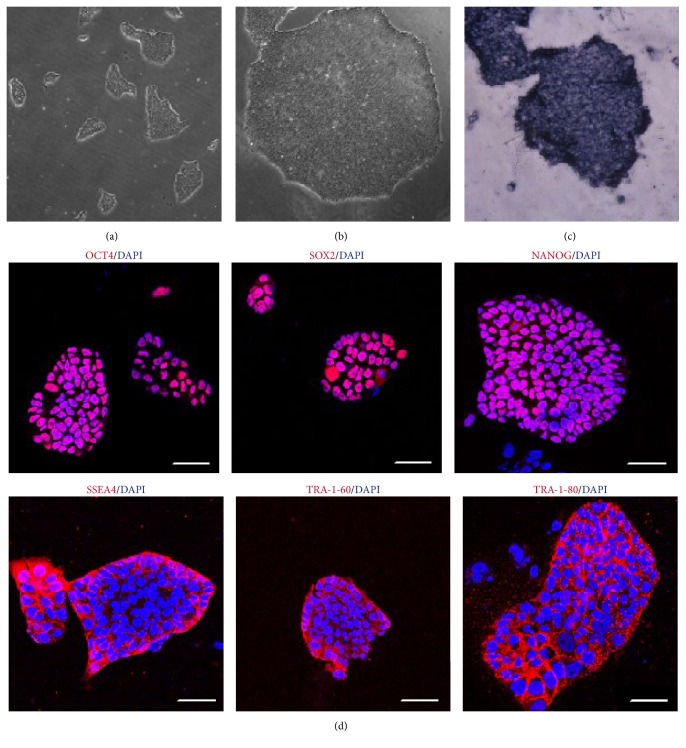
Morphology of NiPS colonies by 1F-OCT4. Low (a) and high (b) magnification of NiPS colonies. 1F-OCT4 human NiPS colonies were stained for alkaline phosphatase (c); immunofluorescent analysis of pluripotency and surface markers (d). The pluripotent markers (OCT4, SOX2, and NANOG) and surface markers (SSEA4, TRA-1-60, and TRA-1-81) were stained with antibodies in human 1F-OCT4 NiPS cells. Nuclei are stained with DAPI (blue). Scale bars, 100 *μ*m.

First we identified markers of human NPCs with immunofluorescent staining. The cultivated human NPCs were stained with antibodies of SOX2 and Nestin, the key transcription factor and surface marker, by immunofluorescent analysis. It was observed that SOX2 and Nestin were endogenously expressed in most human NPCs (Figure 1). Then, we transfected 1F-OCT4 to human NPCs by electroporation. Three days after the transfection, the cells were plated at about 1 × 10^5^ cells per well in a 35 mm dish precoated with vitronectin. As seen under phase contrast microscopy, the cell colony did not show up until 15 days after transfection (Figure 1(b)). In the following days, the typical colonies appeared, flat and compact, like human ES cells in morphology (Figure 2(b)). We called these colonies NiPS cells. Typically, 100–200 1F-OCT4 colonies could be generated from 1 × 10^5^ NPCs (reprogramming efficiency: 0.1–0.2%).

### 3.2. The Identification of Pluripotent Markers of NiPS Cells

These NiPS cells could be passaged by EDTA and exhibited positive features by alkaline phosphatase (AP) staining (Figures 2(a), 2(b), and 2(c)). Immunofluorescence staining confirmed human NiPS cells from 1F-OCT4 transfection widely expressed human pluripotent cell markers, including alkaline phosphatase, OCT4, SOX2, NANOG, SSEA4, TRA-1-60, and TRA-1-81 (Figure 2(d)). These results demonstrated that human iPS cells could be generated from human NPCs by a factor OCT4 alone.

### 3.3. Gene Expression Profiling, DNA Methylation, and Differentiation* In Vivo*

**Figure 3:**
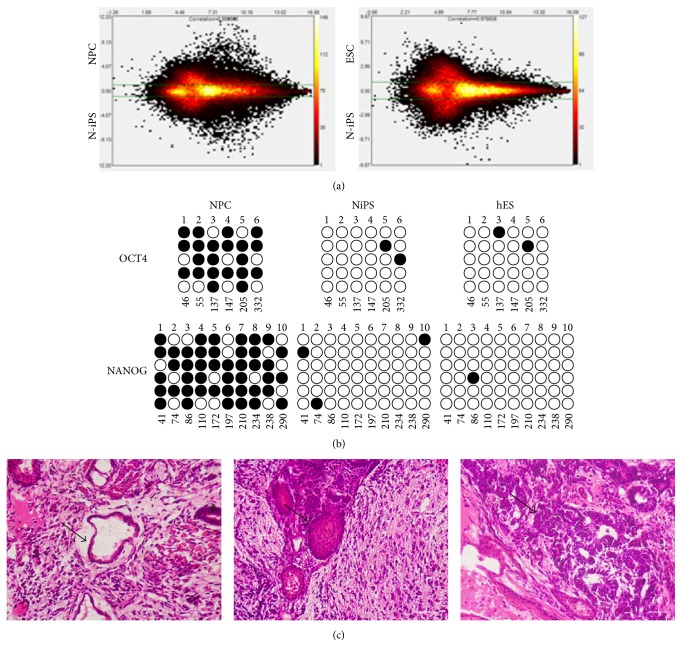
Analyses of DNA methylation and gene expression profiling in NiPS, NPC, and hES cells. (a) Analysis of gene expression profiling: the MvA plot is a comparison plot comparing two microarrays. The *y*-axis is displayed on a log2 scale with green threshold lines for two-fold changes. The lower parts of MvA plot represent signals of NiPS cells, while the upper parts represent signals of NPC or hES cells. The color coding of the plot indicates the density of probes represented by that data point; (b) bisulphite sequencing analysis of OCT4 and NANOG promoter regions in human NSCs, 1F-OCT4 human NiPS clones, and human ES cells; (c) Hematoxylin and Eosin (HE) stain of teratomas generated from 1F-OCT4 derived iPS cells injected subcutaneously into immunocompromised SCID mice. Structures derivative of all three germ layers could be identified. (i) Neural tissue (ectoderm, left), (ii) cartilage (mesoderm, middle), and (iii) gut-like epithelium (endoderm, right).

The global gene expression profiling of NPCs, NiPS, and hES cells was performed by Affymetrix Human U133 plus 2.0 microarrays. The comparison of two microarrays was performed by Affymetrix Expression Console Software 1.3. We use MvA plots to compare expression profiling of NiPS and NPC and NiPS and hES cells. Pearson's correlation (*R* ^2^) of NiPS and NPC and NiPS and hES cells was 0.87 and 0.97, respectively. The result indicated expressions of NiPS cells are more similar to hES cells than those of NPCs (Figure 3(a)). The hierarchical clustering analysis on human NPCs, ESCs, and NiPS cells showed the similar result (see Supplementary Figure  S1 in Supplementary Material available online at http://dx.doi.org/10.1155/2016/4729535). To testify epigenetic status in reprogrammed cells, we used bisulphite sequencing analysis to determine the degree of DNA methylation of the OCT4 and NANOG promoters (Figure 3(b)). Similar to human ES cells H1, both promoter regions were demethylated in 1F-OCT4 human NiPS cells relative to the cultivated human NSCs or NPCs. Our results showed a single transcription factor, OCT4, could reprogram human NSCs into iPS cells that are very similar to human ES cells at the promoters of two genes.

The pluripotency of both NiPS cells was also tested* in vivo*. Eight weeks after injection of 1 × 10^6^ NiPS cells into the testis of SCID mice, tumor mass was isolated and analyzed by immunostaining. As shown in Figure 3(c), the tumor tissues contained neural tissues (ectoderm, left panel), cartilage (mesoderm, middle panel), and gut-like epithelium (endoderm, right panel). These results indicated the feature of teratoma for NiPS cells and revealed the pluripotency of these cells.

### 3.4. Survivin Expression Related to Efficiency of Reprogramming

**Figure 4:**
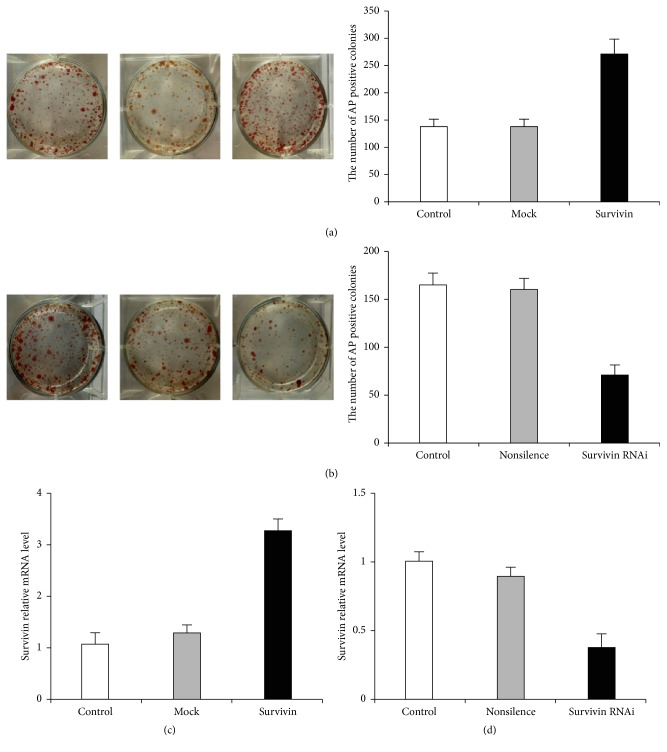
AP positive numbers in Survivin overexpression or Survivin knockdown in the reprogramming of NPCs. (a) AP positive numbers of 1F-OCT4, mock (vector control), and Survivin overexpression on 3.5 cm diameter well from the left to right; (b) AP positive numbers of 1F-OCT4, nonsilence, and Survivin-RNAi on the same size dishes (from the left to right). A representative experiment is shown in the left panels. Counting AP+ colonies in the same experiment, mean values + SD are shown in the right panels. (c) The relative expression of Survivin in mRNA level.

Survivin is an important factor that related to ES cell pluripotency maintenance; we checked Survivin expression manner during ES cell differentiation and reprogramming. We evaluated the effect of 1F-OCT4 infected NPCs with high level or low level of Survivin in the process of NPC reprogramming. At days 12–15 after infection, most alkaline phosphatase-positive (AP+) colonies appeared in the group of 3.5 cm diameter well with Survivin overexpression compared to groups of the control or mock vector. The number of colonies in the group of Survivin overexpression was two-fold compared to the group of 1F-OCT4 control and mock vector (*n* = 3, *p* < 0.05) (Figure 4(a)). On the contrary, low Survivin expression (about 70% reducing) by RNAi leads to a half AP+ colonies formation compared to the 1F-OCT4 control or nonsilence (*n* = 3, *p* < 0.05) (Figure 4(b)). The mRNA relative level of Survivin overexpression group was about 3.5-fold compared with control and mock groups (Figure 4(c)). When Survivin was inhibited by RNAi, the mRNA relative level was decreased 3 times compared with the control and nonsilence groups (Figure 4(d)). These studies suggested that silencing of Survivin reduces iPS cells generation and that the expression of the self-renewal regulator Survivin is absolutely essential for cellular reprogramming.

### 3.5. SOX2 and OCT4 Synergistically Regulate Expression of Survivin

**Figure 5:**
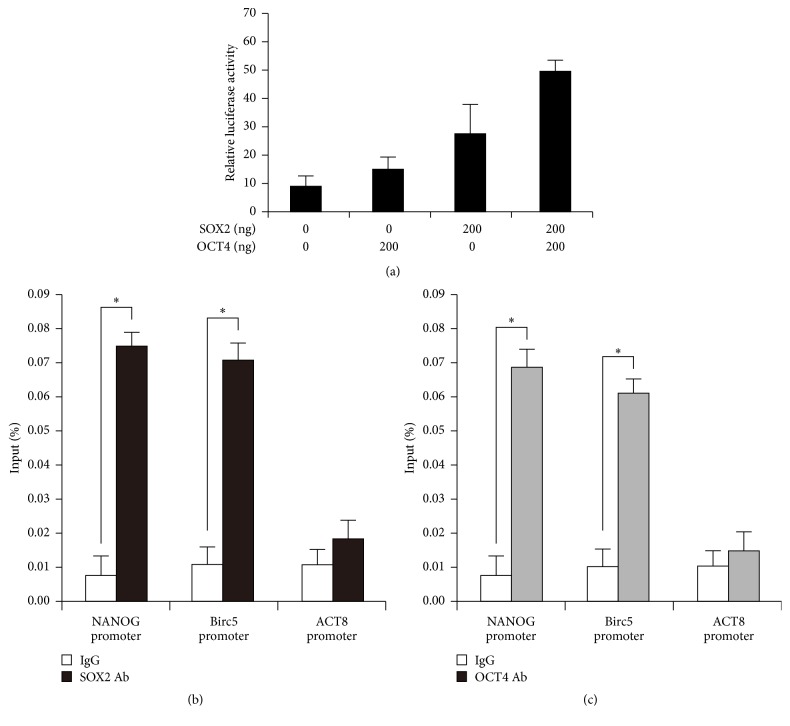
SOX2 and OCT4 synergistically regulate expression of Survivin in ES cells. ChIP-qPCRs were conducted using SOX2 and OCT4 antibodies and primers specific for promoter of NANOG, Survivin (Birc5), and the *β*-actin genes. DNA lysates of cells were as input. NANOG was the positive control while *β*-actin was the negative control.

We constructed Survivin promoter sequences and then measured Survivin transcriptional activity in HEK 293 cells upon adding OCT4 and SOX2 plasmids by luciferase assay. The results showed Survivin promoter driving luciferase expression was positively regulated by OCT4 and SOX2. These two transcription factors had synergistic effects in the regulation of Survivin* in vitro* (Figure 5(a)). To find out how did Survivin participates in ES or iPS cell pluripotency maintenance and reprogramming, we did chromatin immunoprecipitation (ChIP) in ES cells. ChIP-qPCR analysis was conducted using SOX2 and OCT4 antibodies and primers specific for promoter of NANOG, Survivin (Birc5), and the *β*-actin genes. The results showed a higher level of enrichment on NANOG promoter as positive control and Survivin (Birc5) promoters in ES cells. However, the promoter of *β*-actin (the negative control) had no enrichment (Figure 5(b)). ChIP analysis revealed the occupancy of SOX2 and OCT4 in the promoters of Survivin. This result indicated SOX2 and OCT4 could regulate Survivin by binding to its promoter synergistically.

### 3.6. Survivin Controls Pluripotency by Regulating *β*-Catenin Protein Stability

**Figure 6:**
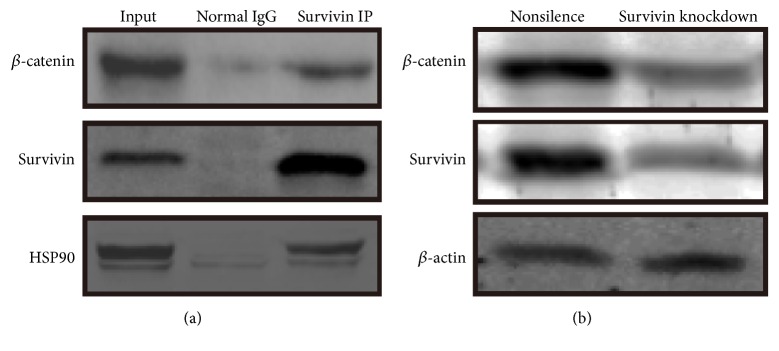
ChIP (chromatin immunoprecipitation) analysis of Survivin in ES (iPS) cells. (a) ChIP of *β*-catenin and Survivin promoter. HSP90 (hot shock protein 90) was the positive control. (b) Survivin-RNAi (knockdown) made *β*-catenin reducing expression by the analysis of Western blot.

It has been shown that activation of the canonical WNT pathway is sufficient to maintain self-renewal of both hES and mES cells [26]. To find out the role of Survivin in reprogramming, we did immunoprecipitation with Survivin in human ES cell. Coimmunoprecipitation (Co-IP) analysis showed *β*-catenin could be pulled down by Survivin (Figure 6(a)). This proved that Survivin could directly interact with *β*-catenin, which is also related to ES cell pluripotency and reprogramming. If Survivin expression decreased by shRNA, the expression of *β*-catenin was also downregulated (Figure 6(b)). These results indicate knockdown of Survivin leads to reduction of *β*-catenin.

### 3.7. Decreased Survivin Expression in the Process of Neural Differentiation of Human ES Cells

**Figure 7:**
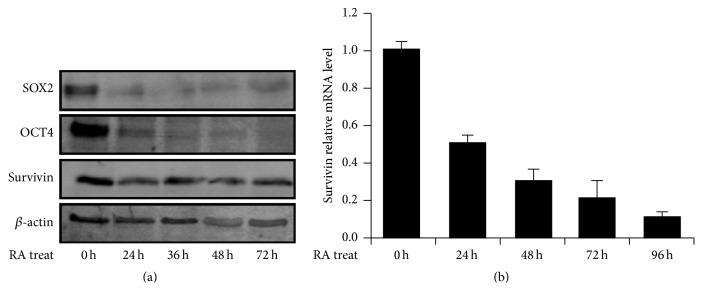
The expression of Survivin was decreased with ES cell differentiation. (a) Expression of Survivin was downregulated with ES cell differentiation with Retinoic Acid (RA). The cells of 0 h, 24 h, 36 h, 48 h, and 72 h were collected for Western blot analysis. The proteins of SOX2, OCT4, and Survivin were analyzed in RA-treated differentiation and *β*-actin as housekeeping control. (b) The change of Survivin mRNA levels in the process of RA-treated differentiation for ES cells. The expressions of mRNA decreased gradually and sharply with prolonged RA-treatment time.

In serum-containing cultures, Retinoic Acid (RA) is essential to induce efficient neural differentiation from ESCs [27]. In the process of ESC differentiation, Survivin was sharply downregulated after 24 h treatment of RA by Western blot analysis. Then it maintained medium expression in the differentiation to neural cells (Figure 7(a)). The pluripotent genes, such as OCT4 and SOX2, were dramatically decreasing in neural differentiation (Figure 7(a)). However, Survivin was gradually decreasing in mRNA level with treatment time prolonged with RA by RT-qPCR (Figure 7(b)). Both RT-qPCR and Western blot analysis showed Survivin expressions were decreased in the process of ES cells differentiation to neural cells. This result indicated Survivin was another factor relative to maintaining pluripotency of human pluripotent stem cells.

## 4. Discussion

The ability of reprogramming of somatic cells provides great potential for developing personalized treatments for diseases and for drug screening [3, 6]. Here we presented that human iPS cells are reprogrammed by a single factor OCT4, from neural progenitor or stem cells with episomal vector. Human iPS cells reprogrammed by OCT4 cultivated with this system appeared to be similar to human ES cells in morphology, surface markers, and the capacity of differentiation to three embryonic germ layers [10]. The formulated and feeder-free mediums were advantageous for NSC reprogramming and experiment repeats. Episomal vectors could be subsequently removed from cells by culturing them (gradual loss of cellular episomal vectors from proliferating cells). Generation of patient-derived iPS cells serves as a great source for neurodegeneration therapies [28]. Reproeasy and PSCeasy medium, which were designed and developed by Cellapy Biotechnology Corporation, are mediums of complete, chemically defined formulation designed for feeder-free reprogramming, maintenance, and expansion of both human ES cells and iPS cells. This kind of cultivated system with defined ingredients mediums, feeder-free, and Xeno-free ECM to maintain human iPS cells has higher safety for clinical applications.

Our results demonstrated that Survivin was one of the factors to participate in ES or iPS cell pluripotency maintenance. We also found overexpression of Survivin in NPCs could enhance the efficiency of 1F-OCT4 reprogramming to iPS cells. The mechanism of Survivin to maintain pluripotent state of ESCs was that it could interact with *β*-catenin in WNT signal pathway. WNT signals and *β*-catenin were also reported relative to ESC's pluripotency and iPS cells' reprogramming [20, 29]. The WNT3a-conditioned media promoted reprogramming of mouse embryonic fibroblasts (MEF) [30]. The evidence proposed by other groups also supported our observation: nuclear *β*-catenin protected cells from apoptosis during the reprogramming process. Based upon these results, they proposed that WNT/*β*-catenin/CBP signaling leaded to improvement of reprogramming efficiency [31]. So, stabilization of *β*-catenin by Survivin was helpful for maintaining pluripotent state of ES or iPS cells. Other researches showed the inhibition of ubiquitin-proteasome pathway blocked ESC differentiation and enhanced cellular reprogramming through stabilization of c-Myc [32]. So the other possibility for that study was that inhibition of ubiquitin-proteasome system might stabilize the *β*-catenin protein. Here we showed Survivin interacted with *β*-catenin in ES cell, and the mechanism might be due to the fact that Survivin-*β*-catenin interaction could prevent *β*-catenin from degradation.

Differentiation capacity of iPS cells to neural cells had no influence in the formulated-ingredient-medium. The key point of iPS cells in medical application depended on whether there are well-established differentiation systems to generate neural cell types [15]. The iPS cells exhibited diverse properties in different cell cultural system. The cultural system of defined ingredients mediums, feeder-free, and Xeno-free ECM for iPS cells could reduce batch differences in the process of reprogramming. The studies of cell-of-origin indicated that the parental cell could influence the differentiation capacity of the resultant iPS cells [33]. There were some reports that showed iPS cells derived from nonhematopoietic cells (neural progenitors and fibroblasts) retained residual methylation [34–36]. Some evidences showed iPS cells retained a residual transcriptional memory of the somatic cells and provided data in support of inefficient promoter DNA methylation as the underlying mechanism [14, 17]. Such a memory would be the fingerprint of the iPS cell's somatic origin. These original memories may help iPS cells from human NSC in more easy and efficient differentiation to neural cells compared with human fibroblast-originated iPS cells.

## Supplementary Material

The profilings of of NiPS closer to those of hESCs than those of NPCs from clustering analysis.
