# Supplementary material for: Survivin Improves Reprogramming Efficiency of Human Neural Progenitors by Single Molecule OCT4
Source: Stem Cells Int. 2016 Nov 16;2016:4729535. doi: 10.1155/2016/4729535 (PMC5128714; doi:10.1155/2016/4729535)
Supplement: Supplementary file 1 — The profilings of of NiPS closer to those of hESCs than those of NPCs from clustering analysis. [file 4729535.f1.pdf]

1. Hierachical clustering analysis

There were three categories clearly separated by microarray hierachical clustering analysis on expression profilings of human NPCs, ESCs and NiPS cells. The same cell types were clustered together for A1 and A2 (NPCs), A3 and A4 (hESCs), A5 and A6 (NiPS). The profilings of two samples of NiPS were closer to those of hESCs than those of NPCs (original cells) from clustering trees.

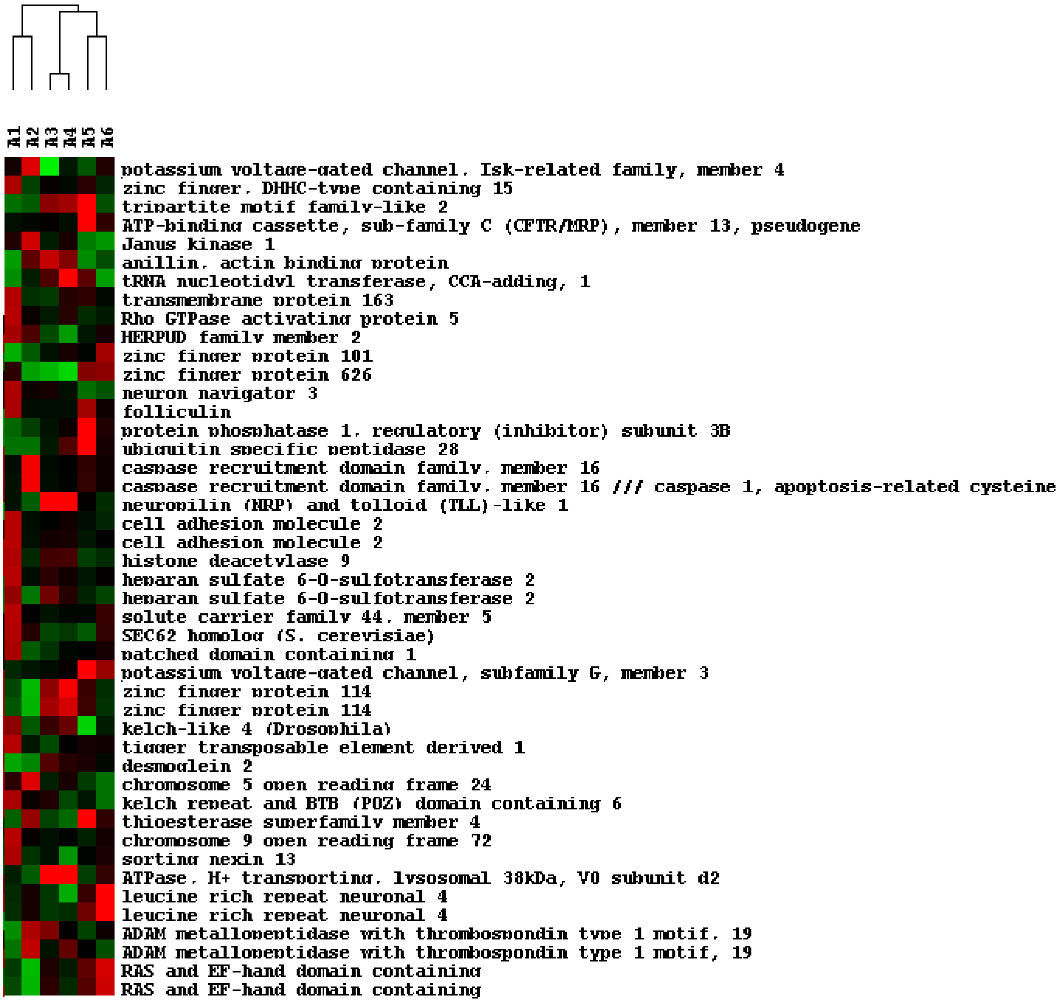

Figure S1. Hierachical clustering analysis on human NPCs, ESCs and NiPS cells. This was a part of hierachical clustering analysis on human NPCs, ESCs and NiPS cells of whole expression profilings. The left of the figure was 6 experiments (from A1 to A6) including 2 samples of NPCs, 2 samples of hESCs and 2 samples of NiPCs. The right of the figure is gene names. In the figure, A1 and A2 represent two samples of NPCs, A3 and A4 represent two samples of hESC H9. A5 and A6 represent NiPS cell 1 and cell 2 respectively.

2. RT-PCR analyses for detection of pCXLE-OCT4 exogenous nucleic acid.

Total RNA of NiPS cells were extracted using the RNeasy Plus Mini kit (Qiagen, Germany) in accordance with the manufacturer’s instructions. The RNA was transcribed to cDNA by transcriptase SuperScriptⅡ (Invitrogen，CA,USA) and anchored Oligo (dT) primers. PCR was performed with specific primers and 30 cycles.

Primers of exogenous hOCT4:

Forward: 5’ACTTCACTGCACTGTACTCCTC3’

Reverse: 5’CATAGCGTAAAAGGAGCAACA3’

Primers of GAPDH (Reference)：

Forward: 5’AACGGATTTGGTCGTATTG3’

Reverse: 5’CACAGTCTTCTGGGTGGC3’

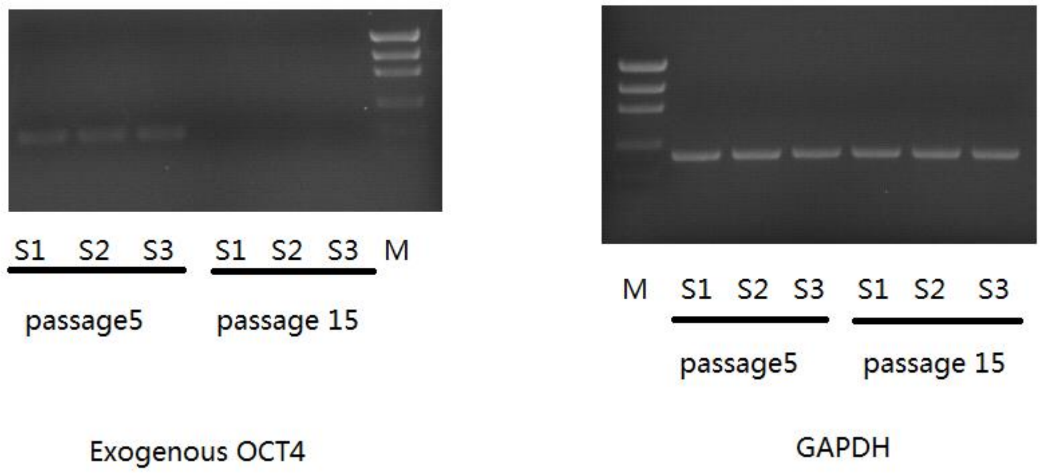

Figure S2 RT-PCR analyses for exogenous OCT4 in NiPS cells of different passages. The left of the figure showed RT-PCR analyses for exogenous OCT4 of NiPS cells from passage 5 and 15. The right of the figure was GAPDH (the reference control) of the same passages of NiPS cells.

The products of RT-PCR were performed electrophoresis by 1% agarose. There were unique nucleic acid bands from 3 clones of passage 5, however these bands were not appeared in passage 15 of NiPS cells. The result indicated the episomal vector was absent in the process of passaging of NiPS cells.

Mrs Xiaoyan Zhang has made some contributions in this project, especially in the process of revising the manuscript. The authors agree to Xiaoyan Zhang as one of co-author. Her email is xyzhang@bjmu.edu.cn
